# Fretting Wear Behavior and Photoelectron Spectroscopy (XPS) Analysis of a Ti/TiN Multilayer Film Deposited on Depleted Uranium

**DOI:** 10.3390/ma11091538

**Published:** 2018-08-27

**Authors:** Shengfa Zhu, Yanping Wu, Zhengyang Li, Liping Fang, Anyi Yin, Jiawei Yan, Fan Jiang, Xiandong Meng, Piheng Chen, Zhenbing Cai

**Affiliations:** 1 Institute of Materials, China Academy of Engineering Physics, Mianyang 621900, China; zhushf-306@163.com (S.Z.); fanglp26@163.com (L.F.); anyiyin@126.com (A.Y.); yanjiawei@caep.cn (J.Y.); ywj-shan@163.com (F.J.); mengxiandong@caep.cn (X.M.); 2 Tribology Research Institute, National Traction Power Laboratory, Southwest Jiaotong University, Chengdu 610031, China; lzy_jiaoda@126.com

**Keywords:** depleted uranium, multilayer film, fretting wear, wear mechanism

## Abstract

Depleted uranium has been widely applied in nuclear energy fields. However, its poor corrosion and wear resistance restrict its applications. A titanium/titanium nitride (Ti/TiN) multilayer film was deposited on a uranium surface to improve its fretting wear resistance. Fretting wear tests were carried out using a pin-on-disc configuration. The fretting behaviors of uranium and the Ti/TiN film were investigated under different normal loads. With the normal load increasing, the mode of fretting wear gradually transformed from slip region (SR) to mixed fretting region (MFR) and then to partial slip region (PSR). It is illustrated that the normal load had an obvious effect on the fretting wear behavior. The friction coefficients of both uranium and Ti/TiN multilayer film decreased with the increase of the normal load. In SR, the main wear mechanisms were delamination and abrasion for uncoated uranium, and delamination and oxidation for uranium coated with the Ti/TiN multilayer film. Photoelectron spectroscopy (XPS) analysis also showed that the Ti/TiN coating was oxidized and formed TiO_2_ during fretting wear. The wear depth of naked uranium was much greater than that of coated uranium, which demonstrated that the Ti/TiN multilayer film could effectively improve the wear properties of uranium.

## 1. Introduction

Depleted uranium (DU) has been widely applied in nuclear energy fields because of its unique nuclear properties. In order to satisfy specific engineering designs, uranium will inevitably be in contact or associated with other materials. The contact interface between uranium and the other material is proved to bear different stresses and relative clearances. During transport and service, uranium will be frequently affected by vibration, causing motion with small displacement amplitude [1,2]. Friction and fretting wear occur when two contacting parts are subjected to small-amplitude oscillatory sliding. Fretting damage can be divided into fretting fatigue, fretting wear, and fretting corrosion, which limit the lifetime of the components [3,4]. The wear debris of uranium are easily corroded in the presence of oxygen and water vapor, because of uranium high chemical reactivity, which presents severe environmental and health hazards [5,6,7].

Surface modification technique is an effective way to enhance the wear-resistant properties of industrial components and has been successfully used to improve the fretting wear resistance behaviors of different materials. Various methods have been used for this purpose, including the use of hard and soft metallic coatings, graphene oxide [8], MoS_2_ [9], nanocomposite coatings [10], and nanolayer coatings [11]. Different coatings have been tested to reduce friction and wear in various applications.

Titanium nitride (TiN) films have attracted much attention to improve the mechanical and tribological properties of materials, and in fact they have been widely applied in the field, for instance for cutting tools and parts of engine [12,13,14,15,16,17,18,19]. The fretting properties of TiN films in varying testing environments [19,20] and at different temperatures [13,16,18] and fretting parameters [12] have been studied. A Ti/TiN multilayer, because of good toughness and tribological behaviour, can significantly improve the wear resistance of the Ti-811 alloy [21]. However, there are few studies on the fretting wear resistance of uranium. The ion-plating technology has attracted tremendous attention due to its low deposition temperature, high deposition rate, and high adhesion strength. It is significant to study the fretting behavior of uranium and a Ti/TiN multilayer film prepared by the arc-ion-plating technology.

In this study, a Ti/TiN multilayer film was prepared on a uranium sample surface by the ion-plating technology. Fretting wear tests were carried out on uranium (indicated as U) and the Ti/TiN multilayer film (indicated as Ti/TiN) under various normal loads. The wear scars were examined by laser confocal scanning microscopy (LCSM), scanning electron microscopy (SEM) and energy dispersive spectroscopy (EDS). The fretting behavior and wear mechanism of the Ti/TiN multilayer film are discussed.

## 2. Experimental Details

### 2.1. Preparation of the Samples

The uranium samples used in this study were abraded, mechanically polished, degreased with acetone and ethanol in an ultrasonic bath, and subsequently dried at room temperature. The sputtering of the Ti/TiN multilayer film was carried out on an ion-plating equipment. The target was high purity Ti (99.99%). The samples were fixed on a continuously rotating planetary holder inside the vacuum chamber. When the base pressure of the vacuum chamber was larger than 5 × 10^−4^ Pa, the samples were further cleaned by argon ion bombardment for 10 min. After pre-sputtering, a Ti interlayer with thickness of about 50 nm was firstly deposited on the uranium substrates to enhance the adhesion strength of the coating. The working pressure was kept at 0.3 Pa. Different N_2_ flows were employed to obtain the Ti/TiN multilayer film. For the Ti layer, argon (99.999%) was introduced into the chamber with a flow of 40 sccm. For the TiN layer, argon (99.999%) and nitrogen (99.999%) were introduced into the chamber with a flow of 10 and 40 sccm, respectively. The alternative deposition of Ti and TiN layers was repeated to obtain the Ti/TiN multilayer film. The deposition time of Ti and TiN was 5 min and 10 min for each layer, respectively. The total deposition time was 1 h. 

### 2.2. Film Characterization

The surface morphologies after fretting wear were examined by scanning electric microscopy (SEM, FEI, Hillsboro, OR, USA). The chemical composition and the elements valence of the Ti/TiN multi-layer film were analyzed by X-ray photoelectron spectroscopy (XPS, Thermo Scientific, Waltham, MA, USA) using the Ka line of the Mg X-ray source (hv = 1253.6 eV). The X-ray gun was 15 kV. The XPS patters were calibrated using C 1s line set at 284.8 eV.

### 2.3. Fretting Tests

**Figure 1:**
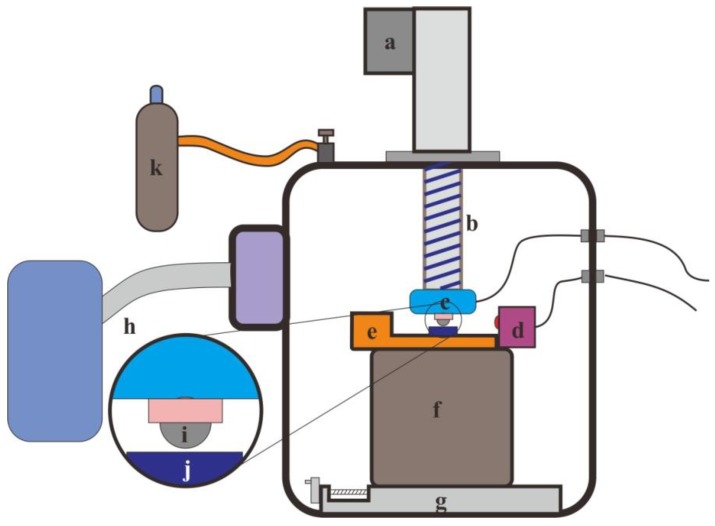
Fretting wear test rig: (**a**) Servo electric cylinder; (**b**) Lead screw; (**c**) Two-dimensional pressure sensor; (**d**) Laser copolymerization sensor; (**e**) Piezoelectric ceramics; (**f**) Base; (**g**) Screw module; (**h**) Molecular pump; (**i**) Counter-body; (**j**) Sample; (**k**) Vacuum chamber.

The fretting tests were carried out on an MFT-6000 machine (CETR, Campbell, CA, USA). A pin-on-disc configuration was employed (Figure 1). The counter-body was a GCr15 ball with the diameter of 12 mm. During the test, the instantaneous displacement and the normal force was monitored and recorded for every cycle. The experimental parameters selected were: displacement amplitude of 20 μm, frequency of 10 Hz, and normal load of 10, 20, 50, and 100 N. The number of cycles was 1 × 10^4^. The fretting tests were conducted in dry conditions at an ambient temperature of 25 °C and relative humidity 50%. Prior to the fretting tests, the samples and counter-body were cleaned with acetone and alcohol. After each test, the morphologies of the wear scars were observed by LCSM (OLYMPUS BX50, Tokyo, Japan) and SEM. The profiles of the fretting scars were assessed using surface profilometry. EDS (FEI, Hillsboro, OR, USA) was used for the element analysis of the wear tracks.

## 3. Results and Discussion

### 3.1. Fretting Regions

**Figure 2:**
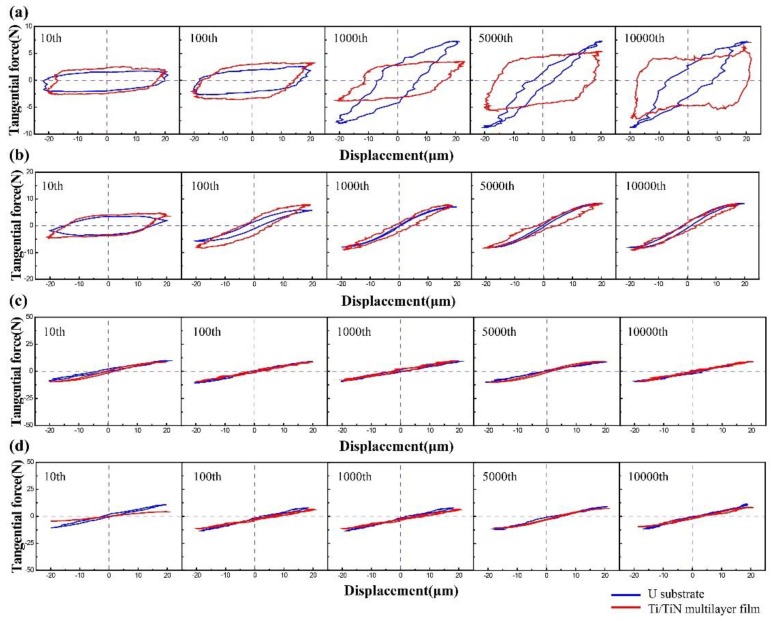
Fretting loops for depleted uranium (DU) and a titanium/titanium nitride (Ti/TiN) film on DU under various normal loads; (**a**) Fn (force of normal loads) = 10 N. (**b**) Fn = 20 N. (**c**) Fn = 50 N. (**d**) Fn = 100 N.

The friction loops of uranium for a normal load of 10, 20, 50, and 100 N, under an imposed displacement amplitude of 20 μm, are shown in Figure 2a–d by blue curves. When the normal load was 10 N (see Figure 2a), the shapes of the F–D curves were quasi-rectangular, and a relative gross slip took place, which was the typical feature of SR in fretting wear [22,23]. The friction loop increased to a higher friction value with the increase of the fretting wear cycles [24]. When the normal load was up to 50 N and 100 N (see Figure 2c,d), the shapes of the F–D curves appeared as elliptical loops in all test cycles. The friction loop shape almost remained the same with the increase of the fretting wear cycles to 10^4^ cycles. This was shown by the flat friction coefficients. Therefore, the fretting processes had run in PSR [21,22]. When the normal load was 20 N, the shapes of the F–D curves were composed of an elliptical loop and a quasi-rectangular loop. A partial slip cycle and a gross slip cycle occurred in the same process. Therefore, the fretting process had run in MFR [22,23].

The friction loops of the Ti/TiN multilayer film for a normal load of 10, 20, 50, and 100 N and a displacement amplitude of 20 μm are also shown in Figure 2a–d, indicated by red curves. The F–D curves shape of the Ti/TiN film on uranium changed from parallelogram to ellipse when the normal load increased from 10 to 20 N, indicating that the fretting process also transformed from SR to MFR. When the normal load was 50 N and 100 N, the shapes of the F–D curves changed to a line in all test cycles. The fretting processes had run in PSR. In short, the transformation trend of the fretting wear behavior of the uranium substrate was in accordance with that of the Ti/TiN multilayer film on U as the normal load increased.

### 3.2. Friction Coefficient

**Figure 3:**
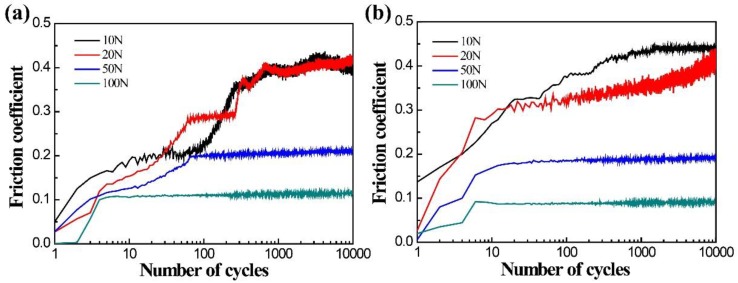
Friction coefficient of DU (**a**) and Ti/TiN on DU (**b**) under various normal loads.

Figure 3 exhibits the evolution of the coefficients of friction (COF) for DU and the Ti/TiN multilayer film with the number of cycles at different normal loads. It is obvious that there was a great difference in COF under various normal loads. The COF decreased with the increase of the normal load for both uranium and Ti/TiN multilayer film. The COF increased from 0.1 at the normal load of 100 N to 0.4 at the normal load of 10 N. The COF was in a steady stage for both uranium and Ti/TiN film in PSR at the normal load of 100 N. The COF of uranium was slightly higher than that of the Ti/TiN film at a high normal load. In the MFR, the COF of the film varied with the cycles. Two stages were observed, including an ascendant stage, corresponding to the COF raising from a lower to a higher level, and a steady stage, characterized by COF values stabilized in a small undulant range, during which an additional third stage causing body wear or a surface roughness change might have occurred. In the SR, the COF of uranium varied with the cycles, and three stages were observed: an initial slowly ascendant stage, a fast ascendant stage, corresponding to the COF raising from a lower to a higher level, and a fluctuant stage, when the COF values descended and ascended repeatedly. The fluctuant stage corresponded to the formation and rejection of debris. 

### 3.3. Fretting Damage and Wear Mechanism

**Figure 4:**
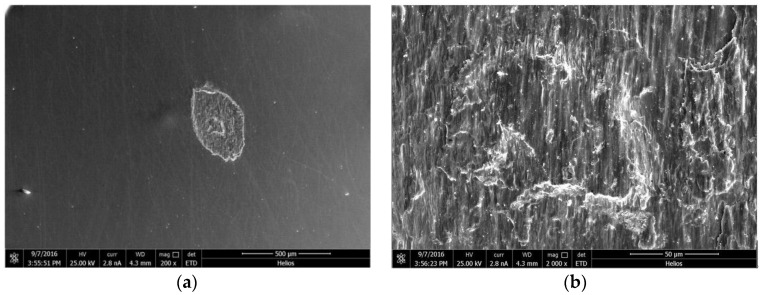

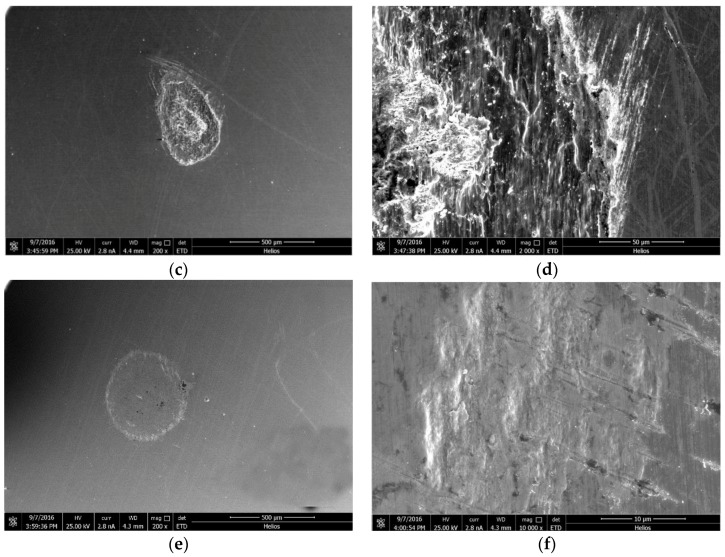
SEM morphologies of the fretting damages on a uranium surface under normal loads of 10 N (**a**,**b**), 20 N (**c**,**d**),100 N (**e**,**f**).

Figure 4 shows images of the typical wear scars of uranium at normal loads of 10 N, 20 N, and 100 N, respectively, and displacement amplitude of 20 μm. As can be seen from Figure 4, the shapes of the wear scars at the normal loads of 10 N and 20 N were nearly elliptic; the shape of the wear scar at the normal load of 100 N was nearly circular. 

In Figure 4a it can be seen that, when the normal load was 10 N, many particles were found on the wear scar. These particles were very small and uniform, which indicated that they had been fully ground in the process of fretting. The particles at the friction interface could easily bind and agglomerate with each other because of their relative motion, resulting in three-body wear and tangential force fluctuation, which was in accordance with the friction coefficient in Figure 3a. Meanwhile, some furrows appeared in the center of the friction interface. A thick wear debris layer covered the contact zones. From the F–D curves, the fretting wear process was in SR, so the main wear mechanisms were delamination and abrasive wear in the SR. When the normal load increased to 20 N, the fretting wear ran in the MFR. A few detached particles covered the contact zone and formed third-body contact. Wear scars were relatively rough with obvious plastic deformation. The center of the wear scars was adhesive, and the edge of the wear scar had a slight sliding area. Lamellate plates were also present in the fretting scars (Figure 4b). Delamination was the main wear mechanism in the MFR. When the normal load was 100 N, only small scratches were observed on the fretting wear area of uranium (Figure 4c). No damage was observed at the contact center. Slight scratches appeared on the contact edge, but there were no wear debris on the wear scar.

**Figure 5:**
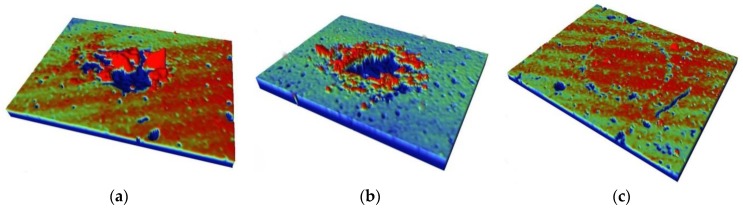
3D profile of the Ti/TiN multilayer film on DU at different normal loads; (**a**) 10 N, (**b**) 20 N, (**c**) 100 N.

The 3D wear profiles of the Ti/TiN multilayer film on uranium are shown in the Figure 5. It can be seen that, at the loads of 10 and 20 N, the wear debris were not discharged completely during the test, so the wear scars were partly bulgy. When the load increased to 100 N, there was just a slightly indentation on the wear surface. The morphology of the contact center was almost unchanged, only the wear edge presented a slightly orbicular damage. 

**Figure 6:**
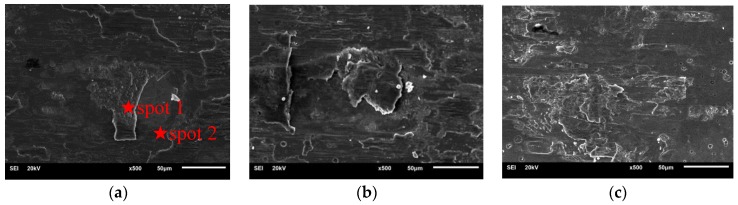
SEM morphologies of the fretting damages on the Ti/TiN multilayer film deposited on uranium under normal loads of 10 N (**a**), 20 N (**b**), 100 N (**c**).

Figure 6 shows images of the typical wear scars on the Ti/TiN multilayer film for a displacement amplitude of 20 μm and normal loads of 10, 20 and 100 N, respectively. From Figure 6a, when the normal load was 10 N, a thick debris layer covered the contact zones, which indicated the wear mechanism was abrasive wear. The wear scars were full of grooves and detached debris, differently from what reported by TiN Zhou [25], who found many fine particles on the wear scars due to the brittle of the TiN film. The reason for this discrepancy is that the soft Ti layer improved the toughness of the Ti/TiN multilayer film, and the layered structure damage clearly appeared at the wear scar because of the different hardness and elasticity modulus of the multi-layer structure. Table 1 shows the element analysis of the wear tracks. In spot 1, the elements were mainly Ti and O with a little of Fe and Cr (mainly coming from the counter-body), which indicated that the detached Ti/TiN debris could be oxides in the process of fretting wear with a normal load of 10 N. In spot 2, the dominant elements were mainly Ti and N from the Ti/TiN multi-layer film. The Ti/TiN multilayer film was degraded as a result of gross sliding. The wear debris on the wear scars of the Ti/TiN multilayer film were significantly fewer than those on uranium. Delamination and oxidation were the main wear mechanisms of the Ti/TiN multilayer film in the SR.

When the normal load was 20 N, the fretting wear ran in the MFR. Lamellate plates also appeared in the fretting scars. Layered structure damage and few wear debris occurred on the wear scars. The substrate material did not emerge on the surface, which indicated that the multilayer film was not worn out. From the EDS spectra [26], at the center of the wear scars, the dominant elements were Ti and N, with a low content of O and Fe. At the edge of the wear scar, the content of Fe and O increased with the decrease of the content of Ti and N. Ti and N appeared in a “W” shape, indicating that the Ti/TiN multilayer film wear was much more severe at the edges than in the center. Delamination was the main wear mechanism of the Ti/TiN multilayer film in the MFR.

It was observed that the Ti/TiN multilayer film had a strong plastic deformation when the normal load was 100 N (Figure 6c). Small damage was observed at the contact center. Lamellate plates appeared in the fretting scars. However, the area of delamination was lower than that at 10 N, and each delamination was adhesive. Only a few wear debris appeared on the wear scar. The reason might be that the fretting ran in the PSR and underwent plastic deformation when the load was 100 N.

**Figure 7:**
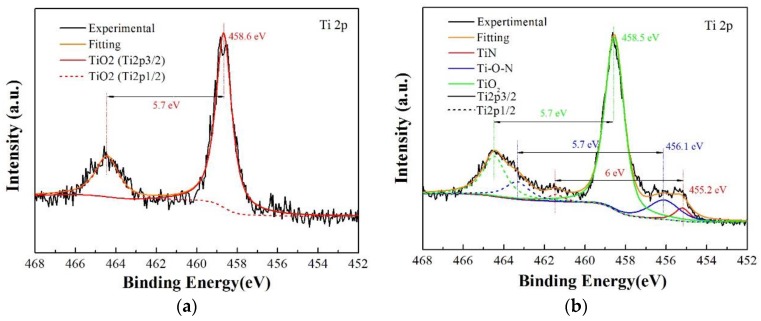
Photoelectron spectroscopy (XPS) Ti2p spectra of the Ti/TiN multilayer film at different normal loads; (**a**) 20 N, (**b**) 100 N.

The XPS Ti2p spectra of the Ti/TiN multilayer film at 20 N and 100 N are shown in Figure 7. In the figure of the XPS Ti2p spectra at 20 N, the peaks at 458.6 eV and 464.3 eV belong to TiO_2_ and are Ti2p3/2 and Ti2p1/2, respectively. In contrast, the XPS Ti2p spectra at 100 N comprise three Ti2p3/2 doublet peaks at 455.2, 456.1, and 458.5 eV, which were assigned to TiN, Ti–O–N, and TiO_2_. It can be seen that the TiN and Ti–O–N peaks appeared at 100 N, because small damage and limited oxidation occurred in the wear scar, partly destroying the Ti/TiN multilayer film. However, at 20 N, the oxidation reaction was severe and the film was oxidized to TiO_2_; therefore, no TiN and Ti–O–N appeared in the wear scar that presented a serious fretting damage.

**Figure 8:**
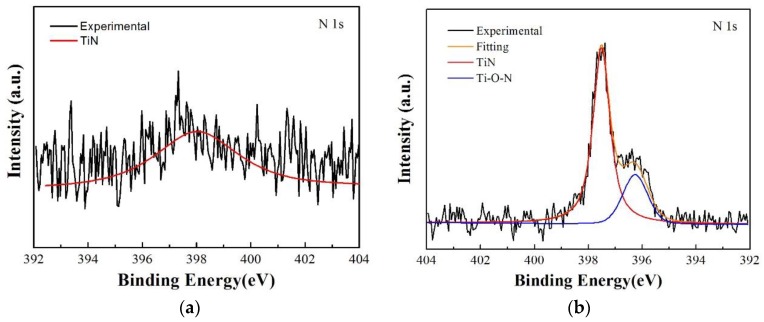
XPS N 1s spectra of the Ti/TiN multilayer film at different normal loads; (**a**) 20 N, (**b**) 100 N.

The XPS N 1s spectra of the Ti/TiN multilayer film at 20 N and 100 N are shown in Figure 8. From the spectra at 20 N, it can be seen that the TiN peak is very weak and no other peak is visible in the spectra. In the spectra at 100 N, the TiN and Ti–O–N peaks can be seen clearly, which indicates that the wear scar was composed of TiO_2_, Ti–O–N, and TiN. The analysis results of N 1s were in accordance with the results of Ti 2p.

### 3.4. Scar Profiles and Wear Rates

**Figure 9:**
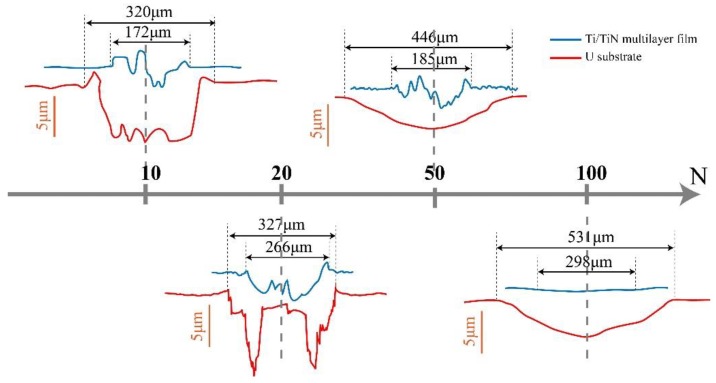
Wear profiles of uranium and the Ti/TiN multilayer film at different normal loads.

The scar profiles were taken along the center line using a profilometer. Figure 9 presents the scar profiles of uranium and the Ti/TiN multi-layer film for various normal loads. In Figure 9, it can be seen that when the normal load was 10 N, the scar presented a “U” shape, typical of a wear scar in SR; when the normal load was 20 N, the scar presented a “W” shape, typical of a wear scar in MFR; the profiles at a high normal load showed a very slight material transfer, which appeared on the counter-body.

Figure 9 shows that the wear depth was larger than the film thickness, when the normal loads were 10 and 20 N. The film was completely removed in the wear scars. At the normal load of 50 and 100 N, the depth of the wear scars in the center was smaller than the film thickness. The film was incompletely removed from the contact zone, although some scratch formed. The protective effect of the film was maintained after 10^4^ cycles. 

The wear depth of the Ti/TiN multilayer film was only of one-tenth of that of uranium, which showed that the Ti/TiN multilayer film improved the wear resistance of uranium.

**Figure 10:**
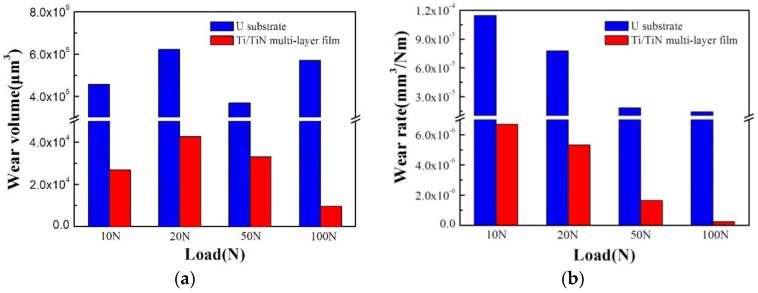
Wear volume and wear rate of uranium and the Ti/TiN film at different normal loads. (**a**) wear volume, (**b**)wear rate.

The wear volumes and wear rates of uranium and the Ti/TiN multilayer film are shown in Figure 10. The wear volume is the amount of material removed from the surface during sliding. The wear rate (*R*) can be calculated using the following equation [11]:

*R* = *V*/(*Fn*·*D*) (1)

where *V* is the wear volume (mm^3^), *Fn* is the normal force applied (N), and *D* is the sliding distance (m).

The wear volume of uranium was irregular, and the maximum wear volume was recorded at the load of 20 N, corresponding to 6.2 × 10^5^ μm^3^. However, the wear volume of the Ti/TiN multilayer film firstly increased and then decreased. This phenomenon was related to the fretting region. From Figure 10b, the wear rates of uranium and the Ti/TiN multilayer film decreased with the load. In generally, the Ti/TiN multilayer film could greatly improve the fretting wear properties of uranium.

## 4. Conclusions

The fretting behavior of a Ti/TiN multilayer film deposited on the surface of a uranium sample was investigated under different normal loads, using a pin-on-disc configuration. The coefficients of friction differed greatly under various normal loads. The friction coefficients decreased with the increase of the normal load for both uranium and Ti/TiN multilayer film. For uranium, delamination and abrasion were the main wear behaviors under different normal loads. In contrast, for the Ti/TiN multilayer film on uranium, delamination and oxidation were the dominant wear behaviors. The XPS analysis results also showed that the Ti/TiN coatings were oxidized and formed TiO_2_ during fretting wear. The wear depth of the uranium substrate was much greater than that of the Ti/TiN multilayer film at the same normal loads, which showed that the Ti/TiN multilayer film could effectively improve the wear properties of depleted uranium.

## Figures and Tables

**Table 1:** Energy dispersive spectroscopy (EDS) analysis results in spots 1 and 2, as indicated in Figure 6a.

Element	Spot 1 Atomic %	Spot 2 Atomic %
O K	75.62	--
N K	--	45.00
Ti K	22.43	47.75
Cr K	0.17	0.77
Fe K	1.79	6.48
